# Spare-part fillet cross-finger flaps: A series of two cases

**DOI:** 10.1016/j.ijscr.2019.06.016

**Published:** 2019-06-13

**Authors:** M.M. Al-Qattan, S.A. Al Mohrij

**Affiliations:** a King Saud University, Riyadh, Saudi Arabia; b Natinal Guard Health Affairs, Riyadh, Saudi Arabia; c Department of Surgery, College of Medicine, King Saud Bin Abdulaziz University for Health Sciences (KSAU-HS), Riyadh, Saudi Arabia

**Keywords:** Cross-finger flaps, Spare-part flaps, Fillet flap

## Abstract

**Introduction:**

The concept of ‘spare-part’ flaps in hand reconstruction is most commonly applied using finger fillet flaps to cover dorsal or volar hand defects. In this paper, we introduce the concept of utilization of spare-part finger fillet flaps in a cross-finger fashion.

**Case reports:**

The technique is demonstrated in two case reports. In both cases, there was one finger that was severely crushed requiring amputation. The bones from that finger were removed and the fillet finger flap was utilized to reconstruct a complex defect in an adjacent digit. Immediate post-operative physiotherapy was employed and flap division was done 17–18 days later. Both patients were satisfied with the outcome and returned back to work as manual workers.

**Discussion:**

Differences between previous techniques / types of cross-finger flaps and the current spare-part fillet cross-finger flap technique are discussed.

**Conclusion:**

We introduce the concept of utilization of spare-part finger fillet flaps in a cross-finger fashion.

## Highlights

• Spare-part finger fillet flaps in a cross-finger fashion have not been described.
• We describe the technique in 2 cases.
• One finger is always severely injured requiring amputation.
• The adjacent finger has a complex defect.
• At final follow up, both patients were satisfied.

## 1 Introduction

Cross-finger flaps were introduced by Cronin in 1951 [1]. Since then, many types and modifications have been described in hand reconstruction.

The term ‘spare-part’ flaps in limb reconstruction means that a flap is taken from a useless or amputated part of the limb to be utilized in the reconstruction of the same limb [2]. For example, coverage of the stump of forequarter amputations in shoulder sarcoma may be done using forearm free flaps harvested from the same amputated limb [2]. In hand reconstruction, the concept of ‘spare-part’ flaps are most commonly applied using finger fillet flaps to cover dorsal or volar hand defects. The bones from the crushed finger is removed maintaining the viability of the soft tissue envelop of the finger based on one or both neurovascular bundles. The fillet finger flap is then used to reconstruct dorsal or volar hand defects [3].

In this paper, we report on two patients who had a severely crushed finger requiring amputation as well as a complex defect in an adjacent viable digit. In both cases, reconstruction was done using the ‘spare-part’ finger fillet flap principle in a cross-finger fashion. This concept has not been discussed in the literature. The work has been reported in line with PROCESS criteria [4].

## 2 Case presentations

**Fig. 1:**
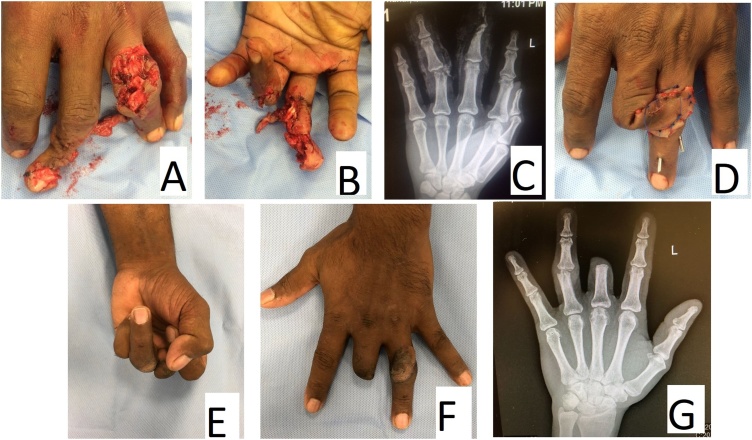
The first case A) and B) The severely crushed middle finger requiring amputation at the level of the proximal interphalangeal joint; and the soft tissue complex defect in the adjacent ring finger. C) X-ray showing the intraarticular fracture of the ring finger. D) Appearance after tendon repair, k-wire immobilization and spare-part fillet cross finger flap reconstruction. E), F), and G) Clinical radiological appearance at 6 months after injury.

The first case (Fig. 1) was a 41-year-old male who sustained a severe crush injury to the left middle finger requiring amputation at the proximal interphalangeal joint. However, the soft tissue envelop of the crushed middle phalanx was still viable based on the ulnar neurovascular bundle. The adjacent ring finger had an intra-articular fracture of the head of the proximal phalanx as well as extensor tendon injury and skin loss over the dorsal aspect of the proximal interphalangeal joint. The tendon was repaired and k-wires were used to stabilized the joint. The soft tissue defect was then reconstructed with a ‘spare-part’ fillet cross-finger flap from the adjacent middle finger. The flap was divided in 17 days and the k-wires were removed 4 weeks after injury. At the final follow-up, 6 months later, the range motion of the ring finger was: 0–80° at the metacarpophalangeal joint, 15°-90° at the proximal interphalangeal joint, and 0–50° at the distal interphalangeal joint. The patient was satisfied with the outcome and return back to his original job as a manual worker.

**Fig. 2:**
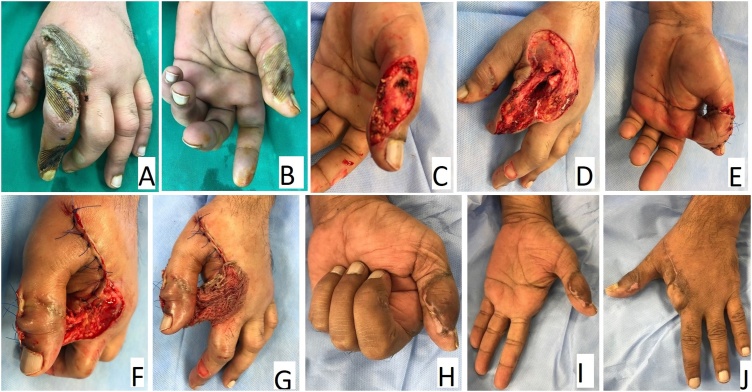
The second case with heat- press injury. A) and B) The severely injured index finger and the injury to the ulnar aspect of the thumb. C) The complex thumb defect following debridement. D) Ray amputation of the index finger in a fillet fashion. E) The spare-part fillet cross-finger flap covering the thumb defect. F) and G) The exposed pedicle of the fillet flap is covered by a skin graft. H), I) and J) Clinical illustrations showing the healed flap and range of motion at 8 months after injury.

The second case (Fig. 2) was a 35-year-old male who sustained a heat-press injury to his left hand. Most of the contact area was to the dorsal aspect of the index finger ray leading necrosis of the dorsal skin and extensor tendons. Since the patient was a manual worker, decision was made to do a ray amputation of the index finger so that he would be able to use his uninjured middle finger as an index finger. The volar skin of the index finger was still viable. Another area of contact to the heat press was the radial aspect of the thumb. Debridement of the burnt area in the thumb resulted in a complex defect exposing the collateral ligaments of the interphalangeal joint and the radial digital nerve. The radial digital artery was thrombosed. Reconstruction of the complex thumb defect was done using a ‘spare-part’ fillet flap from the index finger. A meshed split- thickness skin graft was used to cover the pedicle of the fillet flap. The flap was divided 18 days later. The skin graft was removed from the flap pedicle and the pedicle was then used to cover the amputation stump of the index finger ray amputation. At final follow-up 8 months later, there was full range of motion of the remaining fingers. There was also full range of motion of thumb at the carpo-metacarpal and the metacarpophalangeal joints. At the interphalangeal joint, the range of motion was 0–50°. The patient was satisfied with the outcome and went back to his original job as a manual worker.

## 3 Discussion

The main aim of the current report is to introduce the concept of utilization of spare-part fillet finger flaps in a cross-finger fashion to reconstruct adjacent complex digital defects. Previous authors have utilized finger fillet flaps to reconstruct dorsal or volar hand defects [3,5].

The literature describes three main types of cross finger flaps: the classic, the de-epithelialized, and the adipofascial cross-finger flaps. The classic flap is harvested from the dorsal aspect of the donor finger to reconstruct a complex defect in the volar aspect of an adjacent digit [6]. If the complex defect is on the dorsal aspect of the digit, the cross finger flap is de-epithelialized and turned-over to cover the defect [7]. In the former classic technique, a skin graft is required to cover the donor defect. In the latter technique, skin grafts are required to cover both the donor defect and the flap itself. Cross-finger adipofascial flaps are harvested from the dorsal aspect of the donor finger to cover either dorsal or volar defects in an injured recipient finger [8]. This reduces the donor finger morbidity and skin grafts are only used to cover the adipofascial flap in the recipient finger. In the current technique of cross-finger fillet flaps, no skin grafts are required.

Two major issues are of concern with the use of cross-finger flaps. The first is donor finger morbidity such as painful neuromas, skin grafts instability, cold intolerance, stiffness and poor cosmetic results [9]. In spare-part fillet cross finger flaps, there is no residual donor finger morbidity since the finger is amputated. The second concern is the overall hand stiffness (especially in adults) because of the 3-week period of immobilization until flap division. Over the last 4 years, the senior author (MMA) has adopted a new protocol to overcome this problem [10] and this protocol was applied to the current two cases. Immediate post-operative active and passive mobilization without a splint is done under the supervision of the physiotherapy department. Furthermore, flap division is done at 17–18 days instead of the standard 3-week period.

## 4 Conclusion

We introduce the concept of utilization of spare-part fillet finger flaps in a cross-finger fashion and demonstrate the technique in two cases. The technique has many advantages such as simplicity, reliable blood supply, and lack residual donor finger morbidity.

## Declaration of Competing Interest

None.

## Funding

None.

## Ethical approval

The study was approved by the research committee, National Hospital (Care), Riyadh, Saudi Arabia.

## Consent

Written informed consent was obtained from both patient for publication of this case report and accompanying images. A copy of the written consent is available for review by Editor-In-Chief of this journal on request.

## Authors’ contribution

Both authors contributed significantly and in agreement with the content of the manuscript. Both authors participated in data collection and in writing of the manuscript.

## Registration of research studies

researchregistry4941.

## Guarantor

M M Al-Qattan.

## Provenance and peer review

Not commissioned, externally peer-reviewed.
